# Exploring conservative avenues in subacute subdural hematoma: the potential role of atorvastatin and dexamethasone as lifesaving allies

**DOI:** 10.1186/s41016-025-00393-8

**Published:** 2025-04-02

**Authors:** Tao Liu, Chenrui Wu, Weiwei Jiang, Mingqi Liu, Zhuang Sha, Rongcai Jiang

**Affiliations:** 1 Department of Neurosurgery, Xuanwu Hospital, Capital Medical University, Beijing, 100053 China; 2 Department of Neurosurgery, Tianjin Medical University General Hospital, Tianjin, China

**Keywords:** Conservative treatment, Subdural hematoma, Atorvastatin

## Abstract

**Background:**

Most cases of acute subdural hematoma (ASDH) require emergency surgery; only a few patients can survive without surgery in the early stages and then develop into subacute subdural hematoma (sASDH). However, the optimal conservative treatment has not yet been established for these sASDH patients. Based on our previous studies, atorvastatin plus dexamethasone may be safe and effective for them. This article aims to document such cases and analyze the possible mechanisms.

**Case presentation:**

We selected five patients with sASDH who received a treatment regimen of atorvastatin plus low-dose dexamethasone without surgery. We then observed the clinical and radiological features during treatment and follow-up. The PubMed database and Google Scholar were retrieved for literature regarding the efficacy and safety of conservative treatment in patients with ASDH/sASDH. We extracted information including authors, sample size, gender, number of patients (death, poor prognosis, delayed surgery), and risk factors.

**Results:**

Of the five patients, all patients who refused surgery for various reasons were resolved after treatment with atorvastatin plus low-dose dexamethasone for their conditions. No hematomas recurred or progressed during an at least 6-month follow-up. We identified 6 studies after searching the database; a total of 1374 patients (F:M = 3:7) with ASDH/sASDH received initial conservative treatment. The pooled results showed that 13.1% of patients who initially received conservative treatment deteriorated and required delayed surgical treatment. Of 1374, the overall incidence of poor prognosis was 19.2%, and 7% of patients eventually died.

**Conclusions:**

It is essential to establish an optimal conservative treatment for patients with sASDH who cannot undergo surgery in an emergency for various reasons. Atorvastatin plus dexamethasone may be an alternative treatment in such a subgroup of sASDH, although a randomized proof-of-concept clinical trial is needed.

## Background

Acute subdural hematoma (ASDH) is the most common focal lesion in traumatic brain injury and is associated with high mortality and morbidity. Although guidelines for surgical decision-making in patients with ASDH are widely applied, the level of evidence is low [1, 2]. Some patients in undeveloped countries may miss emergency surgical treatment due to surgical contraindications, mild consciousness disorders, or economic reasons. There are also subjective social factors. For example, patients and their families may request conservative treatment for religious reasons or due to customs specific to certain ethnic groups. In these patients, ASDH may transition to subacute subdural hematoma (sASDH), and conservative therapy should be adapted accordingly. However, no consensus exists on the nonsurgical management of these patients.

Since there are rare guidelines or consensus directing the nonsurgical treatment for sASDH patients, delayed surgery becomes the remedial measure. Pharmacotherapy should be developed to prevent rapid deterioration in sASDH patients who are hesitant about surgery. However, the optimal approach for treating sASDH with medications and evaluating their neurological status or prognosis remains to be elucidated. Furthermore, there is little research concluding on this topic. Given the actual situation in which clinical decisions are influenced by various factors (such as the presence of absolute surgical contraindications or refusal of surgery due to psychological, social, religious, or family factors), it is urgent to explore an effective conservative treatment method for sASDH before surgical intervention becomes inevitable.

Thus far, dexamethasone, angiotensin-converting enzyme inhibitors, celecoxib, tranexamic acid, and traditional Chinese medicines have been studied for the nonsurgical treatment of patients with chronic subdural hematoma (CSDH). However, they have not shown significant efficacy in clinical trials [3, 4]. According to the results of our previously conducted double-blind, randomized, placebo-controlled clinical trial, we have confirmed that atorvastatin is safe and effective for the nonsurgical treatment of CSDH [5]. In another phase II randomized proof-of-concept trial, we found that atorvastatin plus low-dose dexamethasone was more effective in patients with CSDH than atorvastatin alone [6]. Considering that sASDH and CSDH are categorized based on the different formation times of intracranial hematomas and therefore share highly similar pathological and clinical characteristics, we speculate that atorvastatin combined with low-dose dexamethasone may be safe and effective for sASDH patients initially selecting nonsurgical therapy. Thus, we evaluated the efficacy of atorvastatin combined with low-dose dexamethasone in sASDH.

In this study, we reported five patients with sASDH who received the treatment of atorvastatin plus low-dose dexamethasone without surgery (Table 1). There were no recurrences during the treatment and follow-up period. The ethics committee of Tianjin Medical University General Hospital approved this study (No. IRB2023-WZ-024). The patients and their relatives were informed and agreed to this study and publication. All patients received standard doses of atorvastatin (20 mg/day oral) plus low-dose dexamethasone (2.25 mg/day in the first 2 weeks, 1.5 mg/day in the third week, and 0.75 mg/day in the fourth week).

**Table 1:** Summary of five cases of sASDH treated with atorvastatin plus low-dose dexamethasone

Case	Age/sex	Trauma	Medical history	Symptoms	Initial GCS	CT scan finding (hematoma laterality)	Outcome
1	30, F	No	No	Headache, glossolalia, blurred vision	E4V5M6	Mixed density clot (right)	The patient’s symptoms improved significantly and was discharged after 7 days
2	32, F	No	Hemophilia	Unconsciousness, convulsions, headache, pseudosmia	E3V1M3	High-density clot (left)	Consciousness improved significantly after 3 days, and hematoma was completely absorbed after 1 month
3	85, F	Yes	Cerebral infarction, cerebellar atrophy	Unconsciousness	E1V3M4	High-density clot (left)	Consciousness improved significantly after 2 weeks (GCS11) and was discharged 20 days later (GCS15)
4	97, M	Yes	Atrial fibrillation, rectal and bladder cancer	Light coma	E1V1M5	Mixed density clot (right)	Normal communication was achieved after 9 days (GCS14), and the patient was discharged after 25 days
5	35, F	Yes	No	Unconsciousness, vomiting	E4V5M6	Mixed density clot (right)	The patient’s symptoms improved significantly after 5 days

## Case presentation

### Case 1

**Fig. 1:**
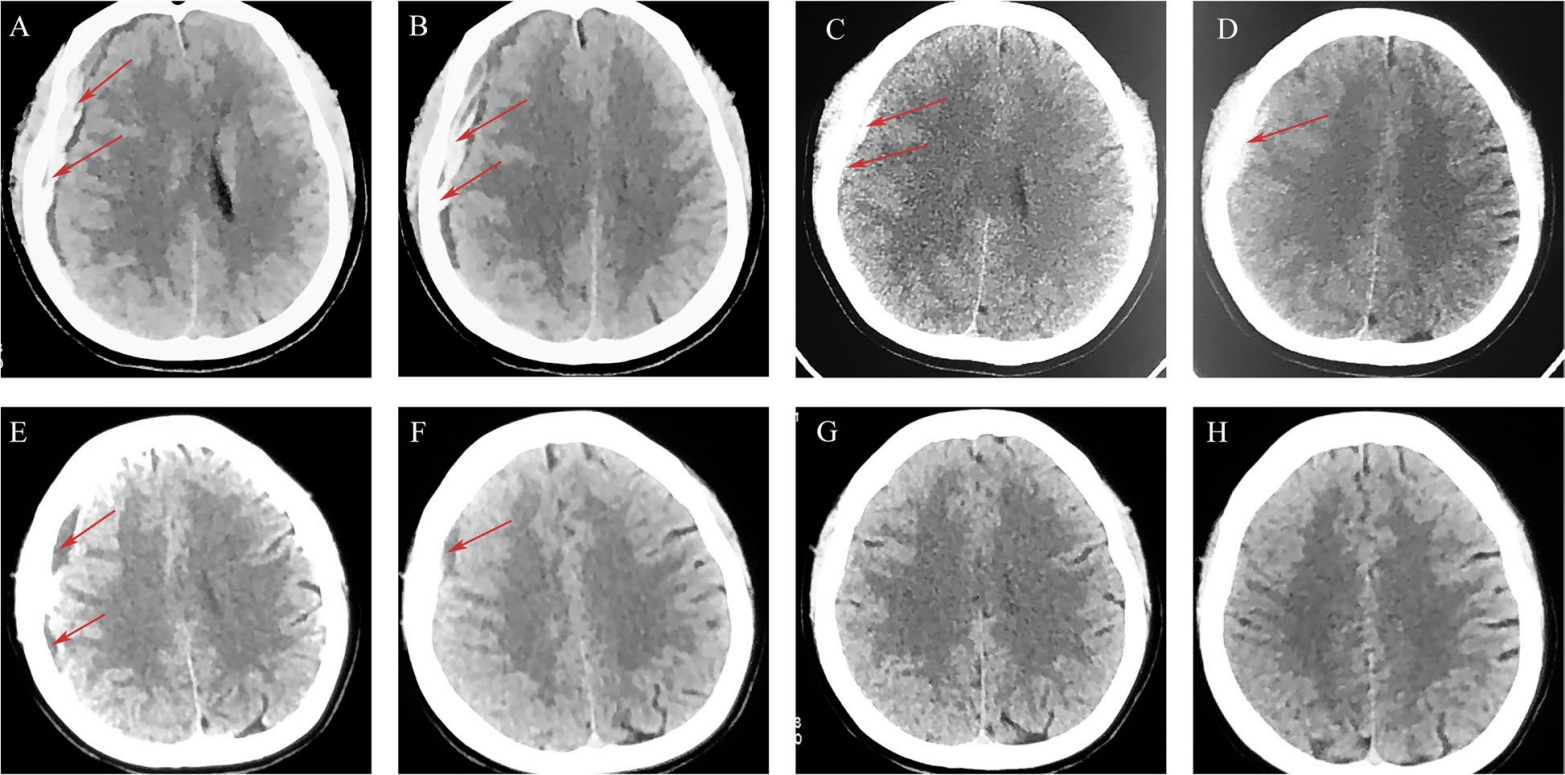
CT scan images for patient 1. ASDH (red arrows) was detected from initial brain CT (**A**, **B**). Follow-up CT scan on the 7th hospital day revealed that the hematoma was reduced (**C**, **D**). After 4 weeks of conservative treatment, the right-side hematoma becomes low-density hydrops (**E**, **F**). Complete resolution of the hematoma was observed after 2 months (**G**, **H**)

A 30-year-old female was initially prepared for eutocia in a local hospital 2 days ago but was switched to a cesarean after 48 h. Seven hours post-operation, the patient experienced transient slurred speech accompanied by headache and blurred vision. Then, she was referred to our hospital. On admission, the patient’s GCS was E4M6V5 with equal and light-responsive pupils. Computed tomography (CT) detected the right subdural hematoma (Fig. 1A, B). The patient strongly requested conservative treatment due to fear of surgery. Atorvastatin (20 mg/day) and low-dose dexamethasone were immediately administered. After 1 week, the patient’s symptoms were significantly relieved. CT showed that the right hematoma had reduced (Fig. 1C, D), and the patient was discharged. Conservative treatment was continued after discharge. After 4 weeks, the hematoma of the patient had significantly absorbed (Fig. 1E, F). The hematoma disappeared, and the patient’s symptoms were completely relieved after 2 months of follow-up (Fig. 1G, H). No adverse drug reactions or hematoma recurrence occurred during treatment and 6 months of follow-up.

### Case 2

**Fig. 2:**
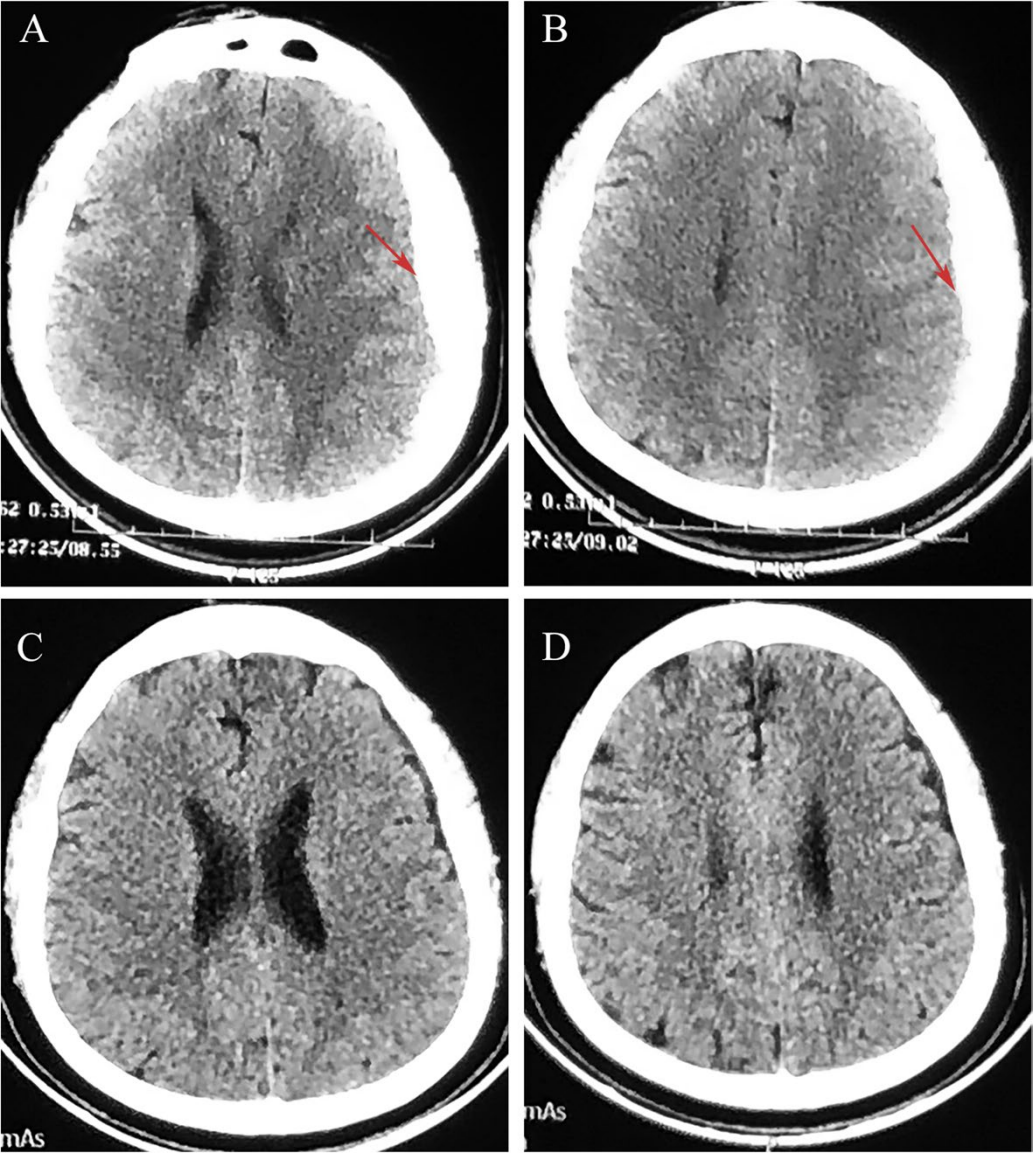
CT scan images for patient 2. Initial brain CT revealed ASDH (red arrows) in the left hemisphere mimicking skull thickening (**A**, **B**). CT scan revealed complete resolution of the hematoma after 2 months (**C**, **D**)

A 32-year-old male with a previous history of congenital hemophilia was treated with an irregular infusion of coagulation factor VIII. Intermittent headache and phantom olfactory symptoms appeared 3 days ago. One day ago, the patient was unconscious and accompanied by convulsions, which lasted for about 1 min. On admission, the patient’s GCS was E3V1M3 following a positive Babinski reflex on the right. The brain CT scan revealed left subdural hematoma mimicking skull thickening (Fig. 2A, B). Considering the possibility of massive blood loss due to difficulty in hemostasis, the patient’s family refused surgical treatment. After 3 days of conservative treatment with atorvastatin and dexamethasone, the patient’s consciousness improved, and the symptoms of headache were significantly relieved. The hematoma was absorbed entirely after 1 month of conservative treatment (Fig. 2C, D), and no drug-related side effects were reported during the treatment period. The hematoma disappeared, and there was no recurrence during a 6-month follow-up.

### Case 3

**Fig. 3:**
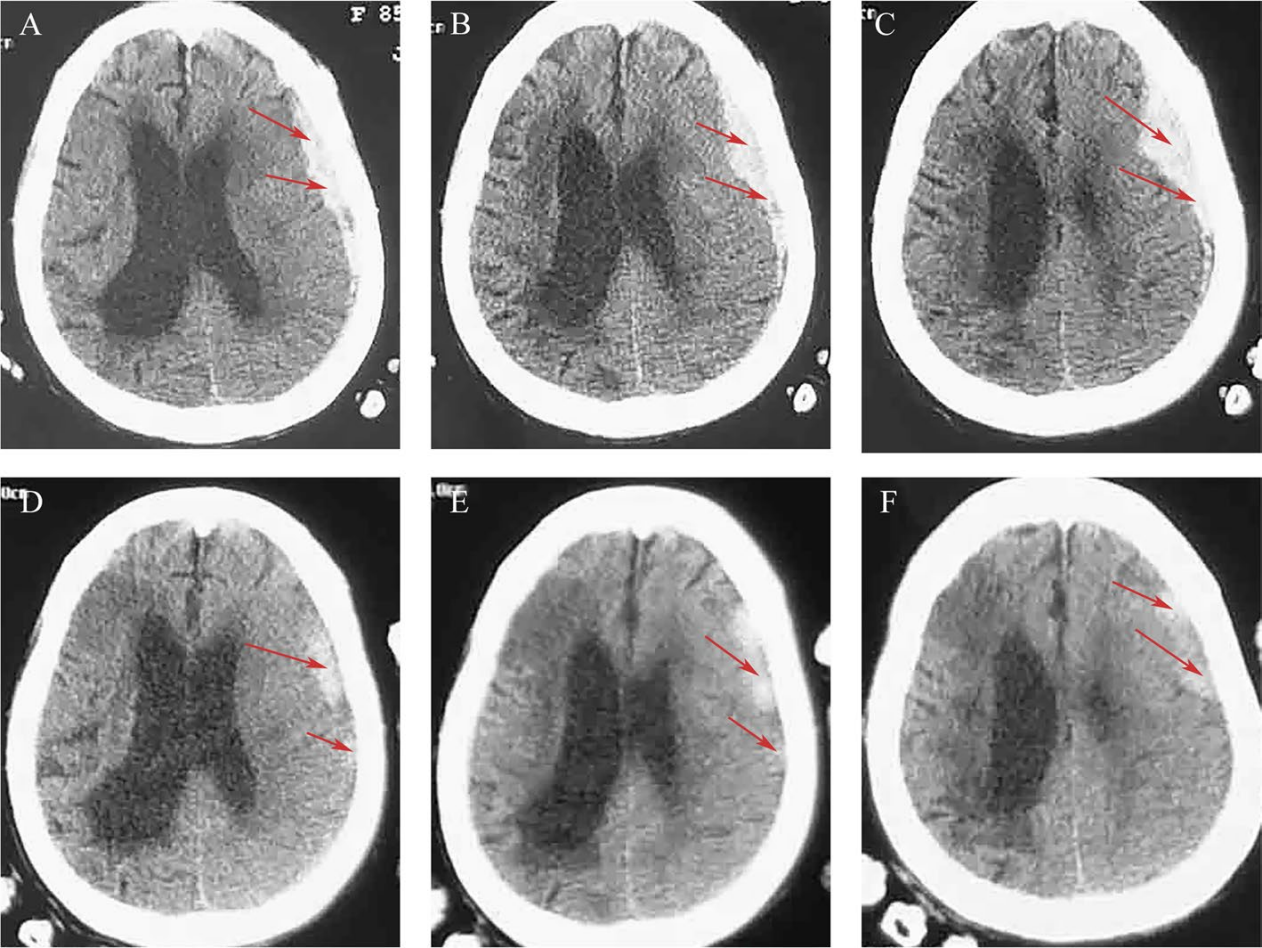
CT scan images for patient 3. Initial brain CT revealed ASDH (red arrows) in the left hemisphere (**A**, **B**, **C**). CT scan revealed significant absorption of the hematoma after 2 weeks (**D**, **E**, **F**)

An 85-year-old woman with a 13-year history of cerebral infarction had been taking aspirin orally for a long time. One day ago, the patient developed an ASDH following head trauma. On admission, the patient’s GCS was E3M5V3, with a disturbance of consciousness (Fig. 3A, B, C). The family members requested conservative treatment due to concerns about the aging patient’s ability to tolerate surgery. After conservative treatment with atorvastatin combined with dexamethasone for 2 weeks, the hematoma was significantly absorbed, and the symptoms were relieved (Fig. 3D, E, F). Due to economic reasons, the patient was discharged after 3 days of the final brain CT scan. It was learned by phone there was no any discomfort during the 6-month follow-up after discharge; therefore, the patient did not undergo a CT re-examination.

### Case 4

**Fig. 4:**
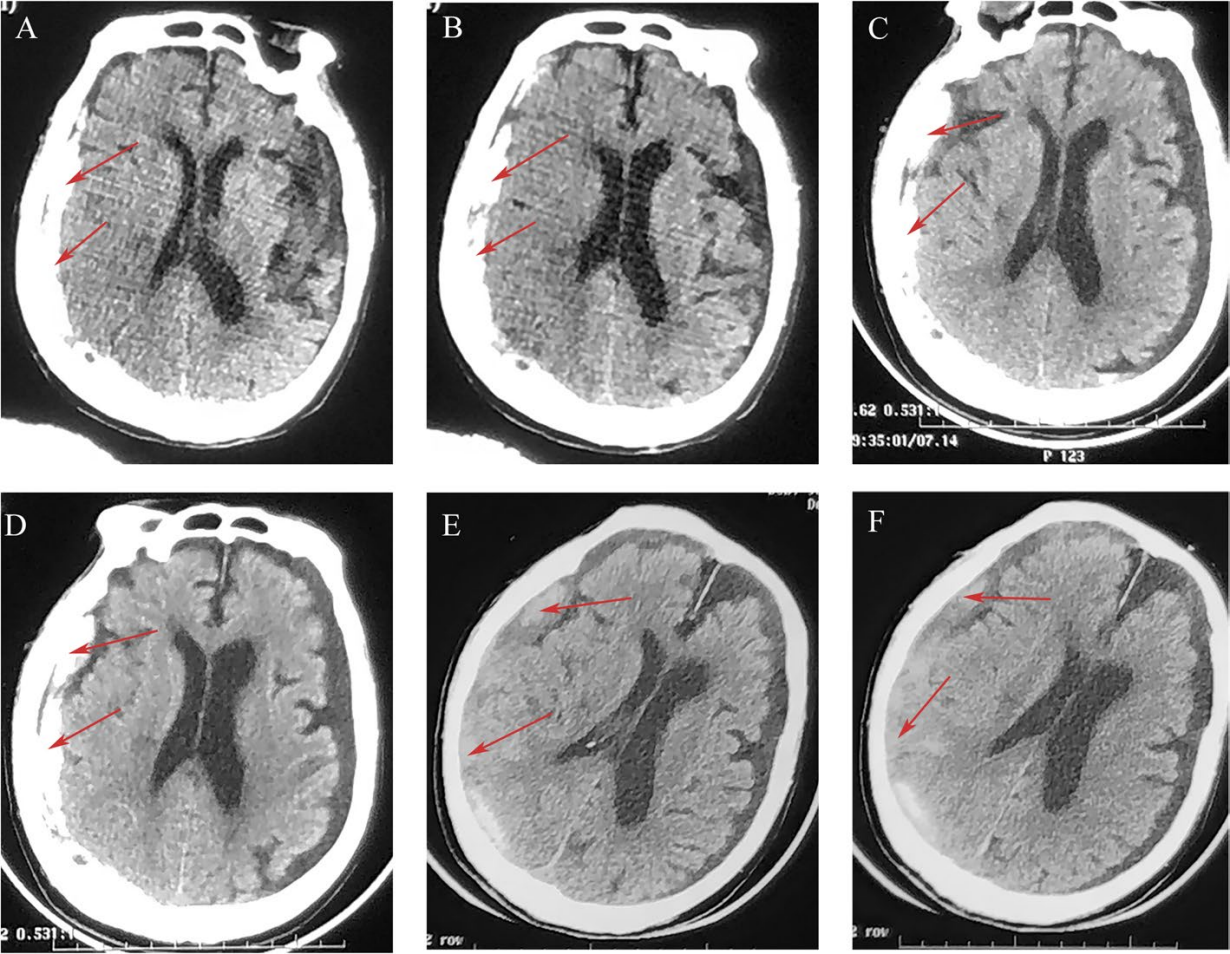
CT scan images for patient 4. Initial brain CT revealed right subdural hematoma (red arrows) (**A**, **B**). Follow-up CT scan on the second hospital day revealed that the hematoma was significantly lessened (**C**, **D**). CT scan revealed significant absorption of the hematoma after 1 week of conservative therapy (**E**, **F**)

A 97-year-old male was presented with disturbance of consciousness and frequent convulsions lasting 2–3 min after trauma 1 day ago. On admission, GCS was E1V1M5. The brain CT scan revealed a right subdural hematoma (Fig. 4A, B). The patient had frequent epileptic seizures during hospitalization. Epilepsy was controlled after treatment with valproate combined with levetiracetam. The family members requested conservative treatment due to the patient’s many underlying diseases. After 2 days of conservative therapy with atorvastatin plus dexamethasone, the patient’s condition was significantly improved with GCS of E3V1M5 (Fig. 4C, D). At 1 week, the hematoma was significantly absorbed with GCS of E4V5M6 (Fig. 4E, F). The patient did not come for re-examination due to the long distance, but during phone follow-up, it was learned that the patient’s clinical symptoms had completely resolved, and his daily life was back to normal. During the 6-month follow-up period after discharge, the patient did not have a hematoma recurrence and could take care of himself as before.

### Case 5

**Fig. 5:**
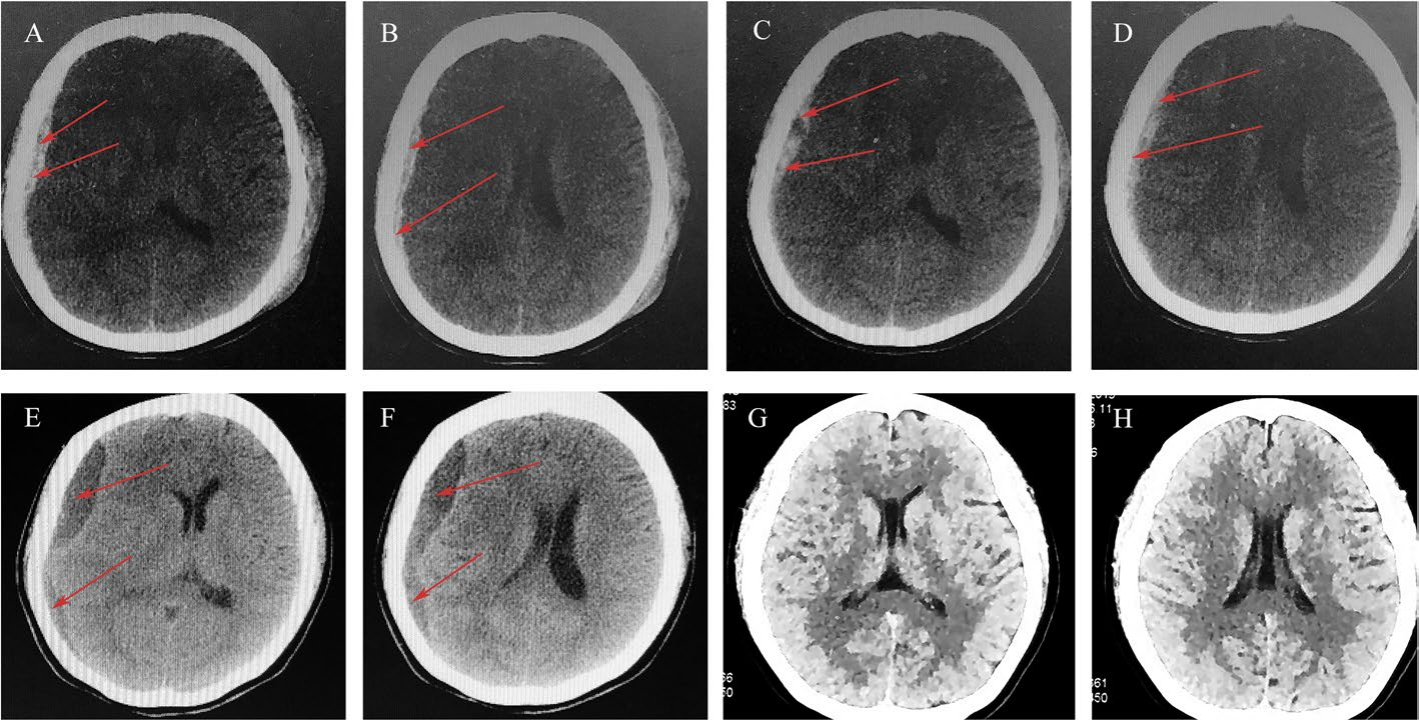
CT scan images for patient 5. Initial brain CT revealed right subdural hematoma (red arrows) (**A**, **B**). CT images obtained after starting atorvastatin plus low-dose dexamethasone therapy. **C**, **D** Day 5. **E**, **F** Day 14. **G**, **H** Six weeks

A 35-year-old female patient was referred to our hospital with a fall history 21 h ago. An unconsciousness lasted for several minutes, followed by spraying vomit, headache, and weakness in the left limb, with GCS of E4M6V5 on admission. Both pupils were equal and responsive to light. CT detected the high-density blood clot (Fig. 5A, B). The patient requested conservative treatment due to economic burden. Atorvastatin plus low-dose dexamethasone was used immediately. After 5 days of conservative treatment, the hematoma size did not change significantly; CT showed mixed-density clot in the right hemisphere (Fig. 5C, D). A follow-up brain CT scan on the 14th hospital day showed low-density clot in the right hemisphere, the headache was significantly relieved, and limb muscle strength was normal (Fig. 5E, F). At 6 weeks, CT showed that the hematoma had been significantly absorbed (Fig. 5G, H). There were no adverse drug reactions or hematoma recurrence during treatment and follow-up.

## Literature review

We searched the PubMed database and Google Scholar for literature regarding the efficacy and safety of conservative treatment in patients with ASDH/sASDH (Table 2). Based on previous studies, we can identify risk factors affecting hematomas’ progression in initially conservatively treated ASDH/sASDH patients [7–12]. These studies were characterized as patients with ASDH/sASDH who received conservative treatment after admission to the hospital. Some patients showed worsening of their condition, requiring surgical decompression. According to the pooled results of previous research, a total of 1374 patients with ASDH/sASDH received initial conservative treatment. The pooled results showed that 13.1% of patients who initially received conservative treatment deteriorated and required delayed surgical treatment. Of 1374, the overall incidence of poor prognosis (poor prognosis, GOS of 1–3; good prognosis, GOS of 4–5) was 19.2%, and 7% of patients eventually died. More than 80% of the patients did not need delayed surgery; this further illustrates that patients with ASDH/sASDH can have a good prognosis with conservative treatment in certain cases. Nevertheless, relatively higher mortality highlights the need to establish an optimal conservative treatment.

**Table 2:** Previous research on progression of hematoma in initially conservatively treated ASDH/sASDH patients

Previous research	No. of patients	Gender (male)	Death	Poor prognosis	Delayed surgery	Risk factors
Paul et al. (2015, USA)	646	69.8%	8.0%	22.2%	42 (6.5%)	Previous fall, alcohol, location (convexity), thickness, midline shifting
Lee et al. (2015, Korea) [8]	177	64.4%	NR	NR	16 (9%)	Age, midline shifting, hematoma depth, Hounsfield unit
Laviv (20 14, Israel) [9]	95	55.8%	20.9%	43.2%	43 (45.2)	IHD, HTN, ACE inhibitor, anticoagulant, clopidogrel, size of SDH
Tomomichi et al. (2020, Japan)	200	50.8%	0	6.5%	17 (8.5%)	Large hematoma brain atrophy, hematoma density
Hyungjoo et al. (2017, Korea)	158	82.1%	7.5%	NR	28 (17.7%)	Age, previous cerebral infarction, increased maximal hematoma thickness, midline shifting, accompanying SAH, low hemoglobin level, high leukocyte count, low glucose level
Kim et al. (2014, Korea) [11]	98	64.3%	0	2%	34 (34.7%)	Thickness, hematoma volume, midline shifting, SAH

*NR* Not reported, *IHD* Ischemic heart disease, *HTN* Hypertension, ACE inhibitor, angiotensin-converting enzyme inhibitor, *SDH* Subdural hematoma, *SAH* Subarachnoid hemorrhage

## Discussion

Surgical treatment is the basis for clinical treatment of ASDH and is an effective way to remove the hematoma, reduce the progression of the condition, and restore nerve function. Drug therapy is not the main focus of them but may be the only option for nonsurgical sASDH. Except for the patients rejecting therapy, most survival sASDH patients who do not suffer from brain hernia could endure the subdural hematoma’s mass effect and even remain awake; therefore, it is an opportunity for them to receive drug therapy. In a tiny proportion of patients with CSDH, we can also observe gradual spontaneous resolution; however, reports of rapid spontaneous resolution of ASDH/sASDH are rare. Our cases belong to benign courses or who refuse active treatment, so no emergency surgery was performed. All cases treated with atorvastatin plus low-dose dexamethasone were resolved, indicating symptomatic sASDH may also benefit from conservative management.

sASDH usually develops from ASDH. The subacute hematoma confined in the subdural cavity contains many broken and dissolved red blood cells, inflammatory cells, and inflammatory cytokines. Actually, the rats with subdural hematoma (SDH) were developed by injecting its autologous blood into the subdural space in our laboratory. It mimics the sASDH/ASDH rather than chronic subdural hematoma because it contains many inflammatory factors and inflammatory cells [13]. Studies have shown that atorvastatin significantly reduces the levels of IL-6, IL-8, and TNF-α in rats with subdural hematoma, thereby alleviating local inflammation, promoting angiogenesis, and contributing to the absorption of SDH in rats [14]. Furthermore, the therapeutic efficiency of atorvastatin plus low-dose dexamethasone for CSDH patients has been preliminarily confirmed [6, 15, 16]. Thus, we adapted atorvastatin plus low-dose dexamethasone for sASDH. These results are consistent with our clinical randomized controlled trials [5].

**Fig. 6:**
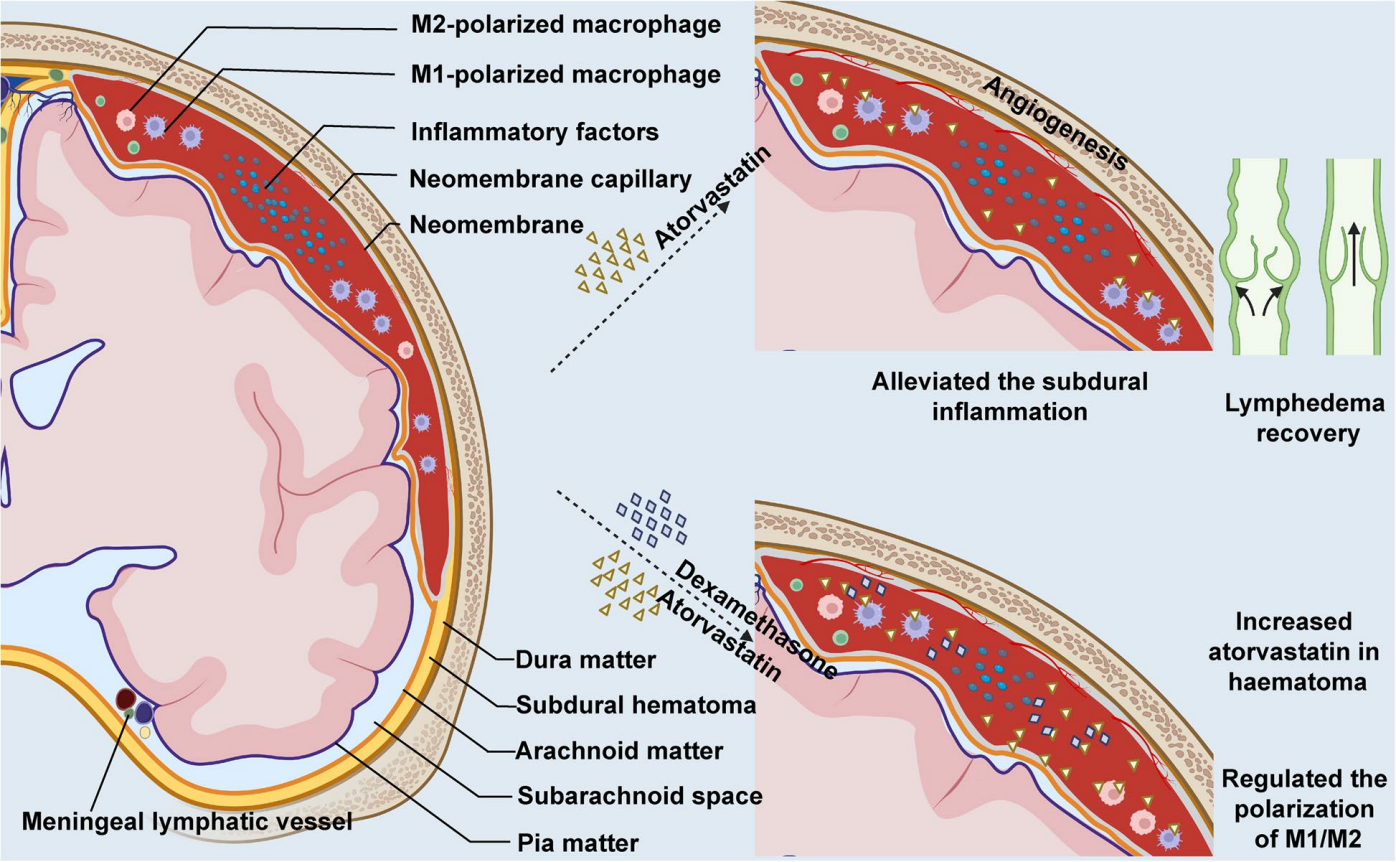
Mechanistic diagram of atorvastatin plus dexamethasone therapy for subdural hematoma

The mechanism of atorvastatin treatment for ASDH is still unclear. Previous studies have suggested that it may be related to inflammation regulation [13, 17]. The discovery of dural lymphatic vessels further elucidates disease progression and provides new therapeutic targets [18, 19]. We postulated the potential mechanism of atorvastatin in the treatment of sASDH is as follows: one through the meningeal lymphatic vessels and the other through the blood vessels of the neomembrane. Studies have found that atorvastatin can promote the proliferation and maturation of blood vessels on the hematoma membrane and increase the absorption of the hematoma. However, due to the sudden onset of sASDH, it is not enough to generate abundant new blood vessels to promote the rapid disappearance of the hematoma; the meningeal lymphatic vessels may also play an important role [20]. ASDH destroys the intima connections of the dural lymphatic vessels, increasing edema and obstructing fluid discharge. Our findings show that atorvastatin can reverse the edema of the dural lymphatic vessels, restore the damaged intima connections, and promote the recovery of dural lymphatic vessel function, thereby promoting the discharge of the hematoma, which is rapid [21]. Our previous research also demonstrated that atorvastatin can be detected in the hematoma fluid of CSDH patients after administration, and that dexamethasone enhances the level of atorvastatin in the hematoma fluid, promotes M2 macrophage polarization, and mitigates inflammation in the subdural cavity (Fig. 6). Therefore, our patients obtain significant improvement in neurological function and cognitive outcomes after short-term conservative treatment.

In summary, the literature review indicated that a portion of ASDH transformed into sASDH; there is no clear description of nonsurgical treatment for these patients, mainly because they did not receive ideal nonsurgical treatment. The high mortality rate of up to 7% indicated an urgent need for suitable nonsurgical treatment. Although the number of cases is small, the application of atorvastatin plus dexamethasone in sASDH, which we first reported, has successfully alleviated symptoms in all cases; this new therapeutic strategy may be a viable option for sASDH.

### Limitation

Our study has some limitations. Firstly, it was an observational study, not a randomized controlled trial. Secondly, this is a single-center study, the cases are all Chinese, and the conclusions drawn are limited. Although we may conduct large-scale randomized controlled trials in the future, there are challenges in developing protocol items, such as inclusion and exclusion criteria, randomization of samples, and ethics.

## Conclusion

In some patients with sASDH transited from ASDH, the nonsurgical treatment strategy of atorvastatin plus dexamethasone is safe and effective. It is recommended to conduct further randomized proof-of-concept clinical trials to verify its efficacy.

## Data Availability

The datasets used and/or analyzed during the current study are available from the corresponding author on reasonable request.

## Abbreviations

ASDH: Acute subdural hematoma
sASDH: subacute subdural hematoma
CSDH: Chronic subdural hematoma
CT: Computed tomography
